# Dual inhibition of EGFR and MET induces synthetic lethality in triple-negative breast cancer cells through downregulation of ribosomal protein S6

**DOI:** 10.3892/ijo.2015.2982

**Published:** 2015-05-04

**Authors:** YONG WEON YI, KYUSIC YOU, EDWARD JEONG BAE, SAHNG-JUNE KWAK, YEON-SUN SEONG, INSOO BAE

**Affiliations:** 1 Department of Nanobiomedical Science and BK21 PLUS Research Center for Regenerative Medicine, Dankook University, Cheonan, Republic of Korea; 2 Department of Oncology, Lombardi Comprehensive Cancer Center, Georgetown University Medical Center, Washington, DC, USA; 3 Department of Nursing and Health Studies, Georgetown University, Washington, DC, USA; 4 Department of Biochemistry, College of Medicine, Dankook University, Cheonan, Republic of Korea

**Keywords:** triple-negative breast cancer, epidermal growth factor receptor, mesenchymal-epithelial transition factor, synthetic lethality, ribosomal protein S6 degradation

## Abstract

Triple-negative breast cancer (TNBC) exhibits innate resistance to the EGFR inhibition despite high level expression of EGFR. Recently, we found that the proliferation of basal-like (BL) subtype TNBC cells is synergistically inhibited by combination of EGFR and PI3K/AKT inhibitors. On the contrary, TNBC cells of mesenchymal stem-like (MSL) subtype are resistant to these combinations. To identify potential synthetic lethal interaction of compounds for treatment of MSL subtype TNBC cells, we performed MTT screening of MDA-MB-231 cells with a small library of receptor tyrosine kinase inhibitors (RTKIs) in the presence of gefitinib, an EGFR inhibitor. We identified MET inhibitors as potent RTKIs that caused synthetic lethality in combination with gefitinib in MDA-MB-231 cells. We demonstrated that combination of a MET inhibitor SU11274 with various EGFR inhibitors resulted in synergistic suppression of cell viability (in MTT assay) and cell survival (in colony formation assay) of MSL subtype TNBC cells. We further demonstrated that SU11274 alone induced G2 arrest and gefitinib/SU11274 combination sustained the SU11274-induced G2 arrest in these cells. In addition, SU11274/gefitinib combination synergistically reduced the level of ribosomal protein S6 (RPS6) in MSL subtype TNBC cells. In addition, knockdown of RPS6 itself, in both HS578T and MDA-MB-231, markedly reduced the proliferation of these cells. Taken together, our data suggest that dual targeting of EGFR and MET inhibits the proliferation of MSL subtype TNBC cells through down-regulation of RPS6.

## Introduction

According to cancer statistics 2014, breast cancer is the top leading cancer in incidence (232,340 cases in USA) with the second highest mortality rate (39,620 death in USA) in women in the United States (1). Triple-negative breast cancer (TNBC), comprising 10–20% of all breast cancers, is a subgroup of breast cancer showing diverse and heterogeneous features with lack of estrogen receptor (ER) and progesterone receptor (PR) expression as well as human epidermal growth factor receptor 2 (HER2) amplification (2,3) and is inadequate to established hormonal therapy and/or HER2 targeted therapy due to the lack of these proteins (4). The TNBC shows poor prognosis due to aggressive biological behavior of tumors as well as earlier involvement of distant metastasis (5). No proven optimistic therapies against TNBCs are established yet and the development of new method on the basis of the weak points of TNBCs is needed (6).

Epidermal growth factor receptor (EGFR) is a member of membrane anchored receptor tyrosine kinase ERBB/HER family comprising of EGFR, HER2, HER3 and HER4 (7,8). The EGFR in normal cells is essential for cell proliferation and survival. Aberrant activation of EGFR by copy number amplification, protein overexpression or point mutation is closely related with unregulated proliferation, malignant transformation, invasion, metastasis and resistance to apoptosis of cancer cells (7,8). Up to 70–80% of metastatic breast cancers shows overexpression of EGFR, but without significant association of HER2 overexpression (9,10). EGFR was found to be expressed at a high level in ~50% of TNBCs and in ~70% of basal-like breast cancers (11,12). Among the groups of TNBC classified by Lehmann *et al*, basal-like 2 (BL2) and mesenchymal stem-like (MSL) subtypes show active EGFR signaling (2). More than 50% of MSL type TNBC is comprised of basal-like features according to intrinsic subtype (13). The germline mutations of BRCA1 and early onset of TNBC is also associated with EGFR activation in breast cancers (14,15). Along with cytokeratin 5/6 as a marker of basal-like breast cancers, the EGFR expression is a marker of poor prognosis regardless of the expression of ER or PR (12,14,16–18). Nevertheless, the results from clinical trials with anti-EGFR combined with platinum or other neoadjuvant agents revealed disappointing results (19–21).

Gefitinib (Iressa) is an orally administrable anticancer agent against EGFR kinase and shows efficacies against various cancers with EGFR activation including breast, lung, colon and other cancers (22–24). Although gefitinib has effects on EGFR activated cancer cells, apparently most TNBC cells with elevated level of EGFR exhibit resistance to EGFR inhibitor treatment. Previously, we found that combination of gefitinib and PI3K/AKT pathway inhibitors synergistically inhibit subsets of TNBC cells *in vitro* (25). On the contrary, regardless of high level expression of EGFR, TNBC cells in MSL subtype including HS578T, MDA-MB-231, and MDA-MB-436 are relatively resistant to these combinations (25). Receptor tyrosine kinase crosstalk, providing surrogate or redundant pathways of cell survival against kinase targeted therapy, is one of the mechanisms of drug resistance (26–31). As an attempt to identify potential receptor tyrosine kinase inhibitors (RTKIs) which induce synthetic lethality in the presence of gefitinib, we performed an MTT screening in MDA-MB-231 cells. We further characterized a MET (mesenchymal-epithelial transition factor) inhibitor SU11274 as a synthetic lethal agent with gefitinib in MSL subtype TNBC cells.

## Materials and methods

### Cell culture and reagents

Reagents for cell culture were purchased from Invitrogen (Carlsbad, CA, USA), Lonza (Basel, Switzerland), or Cellgro (Manassas, VA, USA). HS578T, MDA-MB-231, and MDA-MB-436 were obtained from the Tissue Culture Shared Resource of Georgetown University Medical Center and maintained in the Dulbecco’s modified Eagle’s medium (DMEM) (Lonza) containing 10% heat inactivated fetal bovine serum (Omega Scientific, Inc., Tarzana, CA, USA) and 100 U/ml penicillin/streptomycin (Lonza). SUM149PT was maintained according to the manufacturer’s recommendation (Asterand, Detroit, MI, USA). The viability of cultured cells was monitored by the trypan blue dye exclusion method using the Luna Automated Cell Counter (Logos Biosystems, Gyunggi-Do, Korea). Receptor tyrosine kinase inhibitors were purchased from the following sources: AEW541 from Cayman Chemical (Ann Arbor, MI, USA); AG1024 from Enzo Life Sciences (Farmingdale, NY, USA); BMS-754807 and OSI-906 from MedKoo Biosciences (Chapel Hill, NC, USA); ABT-869, AV-951, BAY 73-4506, BMS-536924, BMS-599626, brivaninb, cediranib, CYC116, E-7080, ENMD-2076, GSK1838705A, GSK1904529A, JNJ-38877605, LDN193189, MGCD265, motesanib, MP-470, NVP-TAE684, OSI-930, PF-2341066 (crizotinib), PHA-665752, SB431542, SB525334, SU11274, Tie2 kinase inhibitor, XL184, and XL880 from Selleck Chemicals (Houston, TX, USA); axitinib, dovitinib, gefitinib, GW-2580, lapatinib, lestaurtinib, masitinib, pazopanib, sorafenib, sunitinib, tandutinib, vandetanib, and vatalanib from LC Labs (Woburn, MA, USA). Genistein and MG132 was purchase from Sigma (St. Louis, MO, USA). Stock solutions of compounds were made in dimethyl sulfoxide (DMSO) and stored at −20°C in small aliquots.

### Synthetic lethal screening

**Figure 1:**
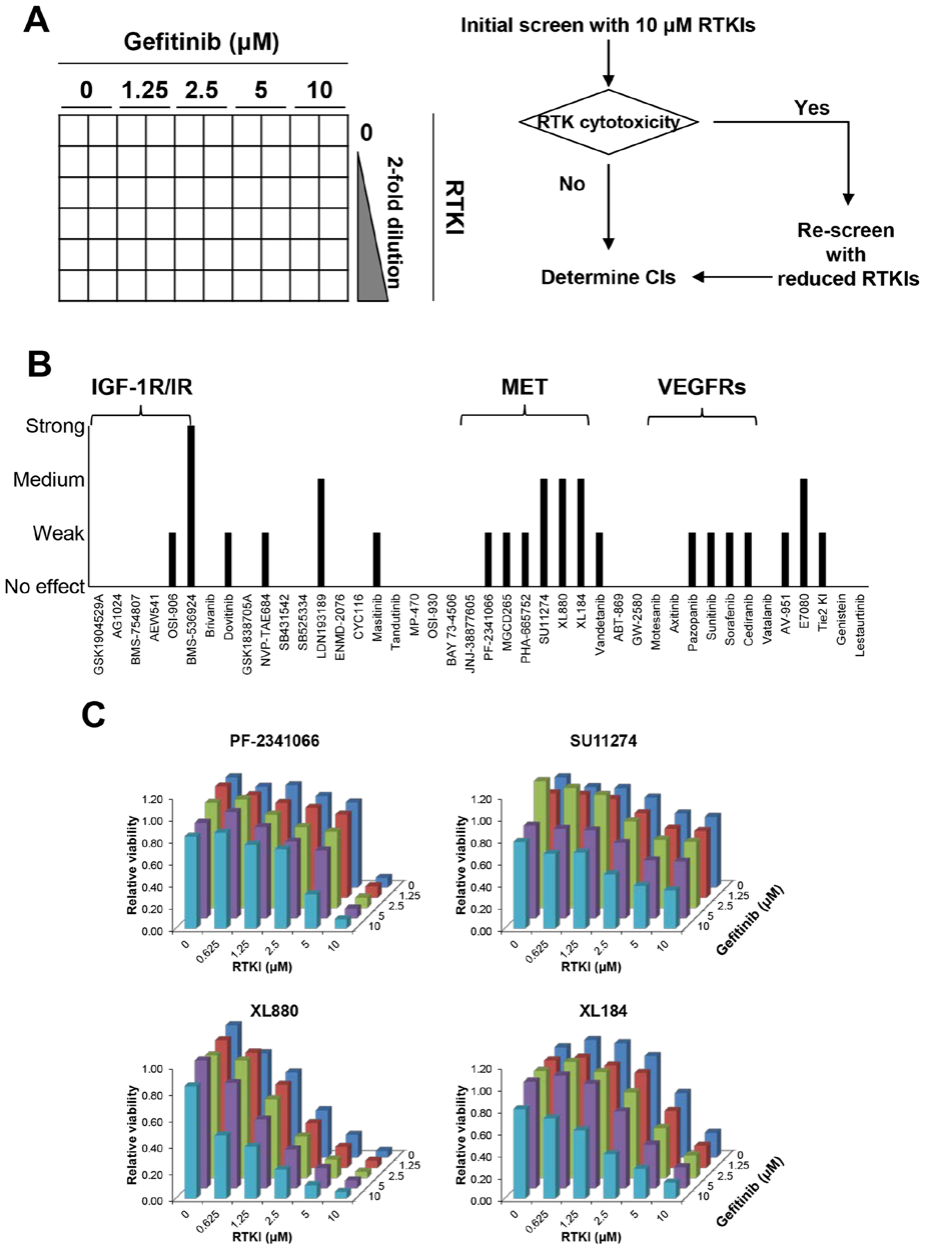
Synthetic lethal screening of MDA-MB-231. (A) Schematic diagram of synthetic lethal screening. (B) RTK inhibitors (RTKIs) which showed synthetic lethality with gefitinib. Strong, medium, and weak synergisms are defined as described in Materials and methods. (C) Representative results of synthetic lethal screening. MDA-MB-231 cells were treated with increasing concentrations of RTKI and gefitinib in duplicates as indicated for ~72 h and viable cells were determined by MTT assay.

MDA-MB-231 cells (2,500 cells/ well) in 96-well plates were treated with increasing amount of gefitinib and increasing amount of RTKIs in duplicates in a 6×5 matrix (Fig. 1A). In an initial screening, the highest concentration of RTKIs was 10 μM. The highest concentrations of RTKIs were reduced when significant reduction of cell viability was observed in single agent treatments. The synergism was determined by calculating classification index (CI) with equation of *A* × *B / AB*, where *A* and *B* are the cell viability with individual agent and *AB* is the cell viability with the combination (32). We further indexed as follows: strong synergism as index 3 when the CI>1.3 at >5 combination points; medium synergism as index 2 when the CI>1.3 at 3 or 4 combination points; weak synergism as index 1 when the CI>1.3 at 1 or 2 combination points. Cell viability was determined at ~72 h after treatment of compounds by MTT (3-(4,5-dimethylthiazol-2-yl)-2,5-diphenyltetrazolium bromide) assay as described previously except for using 4 mg/ml of MTT solution (25,33).

### Clonogenic cell survival assay

Cells were subcultured into 6-well plates with appropriate densities: 500–1,000 cells/well for HS578T and 3,000 cells/well for MDA-MB-231. The day after subculture, the cells were treated with indicated concentrations of compounds for 24 h, and then the cells were supplemented with fresh normal growth media without compounds. The cells were further cultured for 10–14 days after treatment with replacement of fresh normal growth media twice per week. The survived colonies were stained as described previously (34). After intensive washing, the images of colonies were captured by scanner. The relative number of colonies was determined as follows: crystal violet stain of colonies was solubilized by solubilization buffer [1:1 mixture (v/v) of 0.1 M sodium phosphate (NaH_2_PO_4_, pH 4.5) and ethanol] and the observance of solubilized crystal violet was measured by ELx808 microplate reader (BioTek, Winooski, VT, USA).

### Western blot analyses and antibodies

Western blot analyses were performed as described previously (25). Antibodies used in this study were as follows: MET (sc-161), ERK1 (sc-94), and PARP (sc-7150) from Santa Cruz (Santa Cruz, CA, USA); p-EGFR (Y1068) (#2237), EGFR (#4405), p-MET (Y1234/Y1235) (#3123), phospho-AKT (Ser473) (#9271), AKT (#9272), p-ERK1/2 (T202/Y204) (#4370), p-p70 S6K (T389) (#9205), p70 S6K (#9202), p-S6 (S235/S236) (#4856), S6 (#2217) and XIAP (#2045) from Cell Signaling (Danvers, MA, USA); α-tubulin, β-actin, and horseradish peroxidase-conjugated secondary antibodies from Sigma.

### Transfection of siRNA and cell proliferation assay

Transfection of siRNA was performed with Lipofectamine 2000 (from Invitrogen) as described previously (35). In brief, HS578T (0.4–0.6×10^5^ cells/well) or MDA-MB-231 (1.0×10^5^ cells/well) cells in 6-well plates were transfected with 100 pmoles of siRNA mixed with 2.5 μl of Lipofectamine 2000 in serum-free DMEM. After 4-h incubation, cells were supplemented with equal volume of DMEM containing 20% FBS and 200 U/ml penicillin/streptomycin to maintain normal growth condition and further incubated for 3 days. After 3-day incubation, cells were further supplemented with equal volume of DMEM containing 20% FBS and 200 U/ml penicillin/streptomycin and incubated for ≤2 more days. The proliferation of cells was determined by counting viable cells which were stained by acridine orange (AO)/propidium iodide (PI) with the Luna-FLDual Fluorescence Cell Counter (Logos Biosystems). The siRNAs were purchased from Bioneer (Seoul, Korea) with following sequences: control-siRNA, 5′-GAC GAG CGG CAC GUG CAC AUU-3′; and RPS6-siRNA, 5′-GAA GCA GCG UAC CAA GAA A(dTdT)-3′.

### Cell cycle analysis

Cells were treated as indicated and the cells, both attached and floating, were harvested to analyze the cell cycle at the Flow Cytometry and Cell Sorting Shared Resource of Georgetown University Medical Centers described previously (33).

### Statistical analysis

The two-tailed Student’s t-test was applied for statistical analysis. ^*^P<0.05; ^**^P<0.01; ^***^P<0.001.

## Results

### Synthetic lethal screening of RTKIs in MDA-MB-231 cells

Since our previous study identified synergistic effects of EGFR and PI3K/AKT inhibition in a subset of TNBC cells (25), we reasoned that combination of kinase inhibitors with EGFR inhibition might induce synthetic lethality in TNBC cells. We noted that TNBC cells of MSL subtype showed innate resistance to EGFR inhibition. Because overcoming resistance is an unmet need to treat human cancer (36,37), we performed an MTT screening for synthetic lethality with a small library of various RTKIs in MDA-MB-231 cells in the presence of an EGFR inhibitor gefitinib (Fig. 1A). We identified various MET inhibitors (Table I) as potential agents that induced synthetic lethal effects with gefitinib in MDA-MB-231 cells (Fig. 1B). Gefitinib and MET inhibitor combinations synergistically reduced the viable cells in MDA-MB-231 cells in a range of various molar ratios of these drugs (Fig. 1C).

### Cytotoxic effect of EGFR and MET inhibitors in human TNBC cell lines

**Figure 2:**
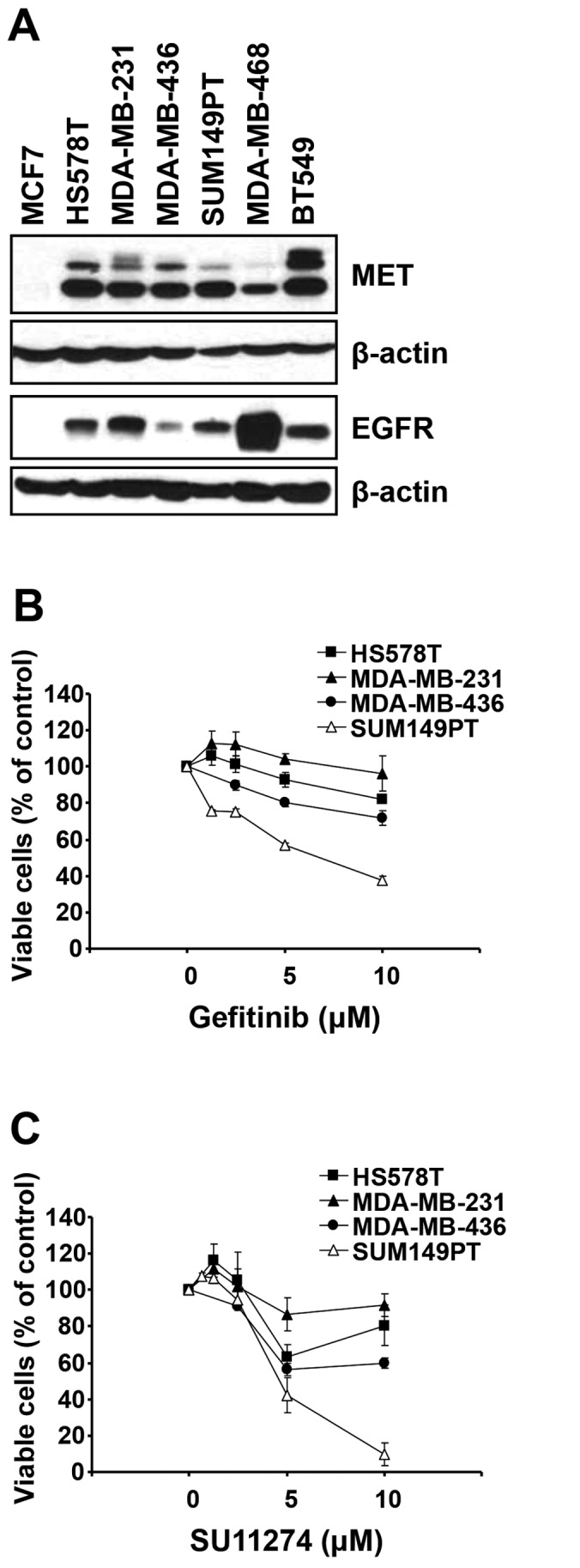
MSL subtype TNBC cells are resistant to either gefitinib or SU11274 in spite of high expression of EGFR and MET. (A) Cell lysates from exponentially growing cells were subjected to western blot analysis with indicated antibodies. β-actin was used as a loading control. (B and C) Cells were incubated with increasing concentrations of gefitinib (B) or SU11274 (C) for ≤72 h and the viable cells were determined by MTT cell viability assay. Data are presented as mean ± SEM from three independent experiments performed in triplicate.

One distinct feature of TNBC is overexpression of EGFR (6,11,12). In addition, a recent study reported that high level expression of MET is an adverse prognostic factor in TNBC patients (38). We determined the level of these proteins in a set of TNBC cell lines by western blot analysis. As reported previously (25), the level of EGFR was high in all TNBC cell lines tested compared to the luminal breast cancer cell line MCF7 (Fig. 2A). The level of MET was also higher in TNBC cells than in MCF7 (Fig. 2A).

Since the levels of both EGFR and MET are elevated in human TNBC cell lines, we further determined the cytotoxic effect of EGFR and MET inhibitors as a single agent toward four different human TNBC cell lines. The cells were treated with the EGFR inhibitor gefitinib or the MET inhibitor SU11274 (39) for ~72 h and the viable cells were determined by MTT cell viability assay. Consistent with a previous report (25), three cell lines (HS578T, MDA-MB-231 and MDA-MB-436) of MSL subtype were relatively resistant to gefitinib compared to a BL2 subtype cell line SUM149PT (Fig. 2B). Gefitinib reduced the viable SUM149PT cells in a dose-dependent manner. On the contrary, the effect of gefitinib was limited on three cell lines of MSL subtype. Additionally, three MSL subtype cell lines were more resistant to SU11274 than SUM149PT cell line (Fig. 2C). Notably, near complete loss of viable cells was observed in SUM149PT cells treated with 10 μM of SU11274, while the effect of SU11274 was less potent toward HS578T, MDA-MB-231 and MDA-MB-436 cells.

### Synergistic cytotoxic effect of EGFR/MET inhibitor combination in human TNBC cell lines

**Figure 3:**
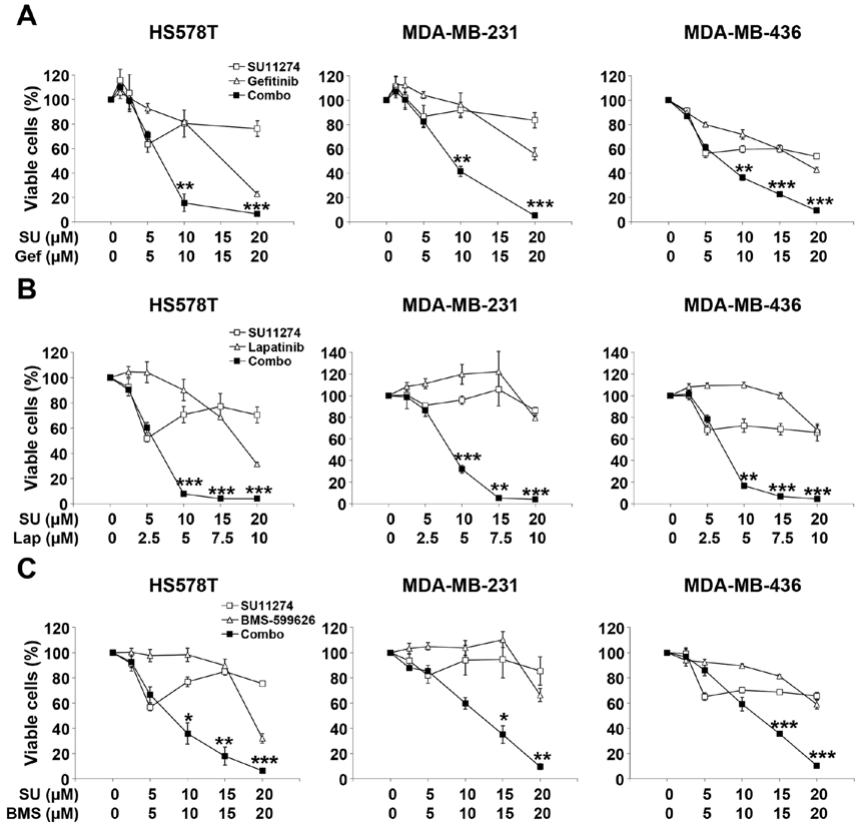
Combination of EGFR inhibitors with SU11274 induces synthetic lethal effect in MSL subtype TNBC cells. Cells were treated with increasing concentrations of compounds as indicated for up to 72 h and the viable cells were measured by MTT assay. Data are presented as mean ± SEM from three independent experiments performed in triplicate. SU, SU11274; Gef, gefitinib; Lap, lapatinib; BMS, BMS-599626. ^*^P<0.05; ^**^P<0.01; ^***^P<0.001.

Since MET inhibitors were identified as potent synthetic lethal agents in combination of gefitinib, we further determined whether the combination of EGFR/MET inhibitors has any beneficial effect in treatment of human TNBC cell lines of MSL subtype. Three types of TNBC cells were treated with increasing concentrations of both EGFR (gefitinib) and MET (SU11274) inhibitors for ~72 h and the viable cells were determined by MTT assay. Combination of gefitinib and SU11274 with fixed molar ratio of 1:1 markedly reduced the viable cells in HS578T, MDA-MB-231, and MDA-MB-436 cells (Fig. 3A). In addition to gefitinib, lapatinib and BMS-599626 (EGFR/HER2 dual inhibitors) also showed marked synergism with SU11274 (Fig. 3B and C). These results suggest that combination of EGFR/MET inhibitors synergistically reduces the cell viability of MSL subtype cell lines of human TNBC.

### Gefitinib/SU11274 combination reduces the survival of human TNBC cell lines

**Figure 4:**
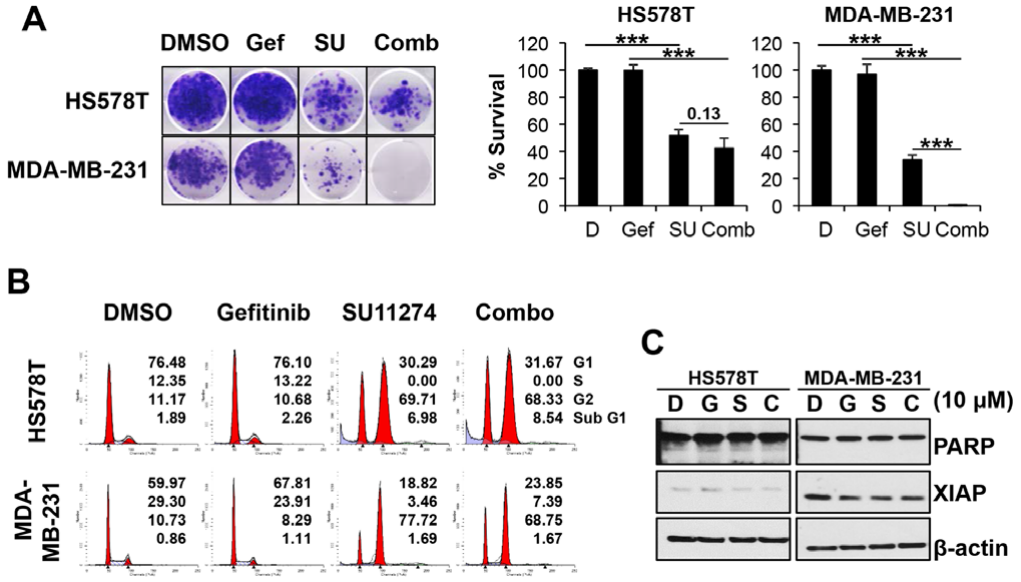
Gefitinib/SU11274 combination reduces survival of TNBC cells through inducing G2 arrest. (A) Cells were treated with compounds (2.5 μM for HS578T or 10 μM for MDA-MB-231) for 24 h and further cultivated for 10–14 days in normal growth media. The survived colonies were stained as described in Materials and methods. Left, representative images from two independent experiments performed in triplicate are shown. Right, relative amount of survived colonies were determined as described in Materials and methods. Gef, gefitinib, SU, SU11274; Combo, combination. ^***^P<0.001. (B) Cells were treated with 10 μM compounds as indicated for 24 h and the cell cycle distribution was determined by FACS analysis. (C) Cells were treated as indicated for 24 h and western blot analysis was performed with indicated antibodies. β-actin was used as a loading control.

The effect of gefitinib/SU11274 combination was further evaluated by clonogenic cell survival assay. TNBC cells were subcultured in 6-well plates in an appropriate density and treated with drug combinations for 24 h. After wash out of drugs, cells were further cultivated in normal growth media. As shown in Fig. 4A, gefitinib alone could not suppress the number of survived colonies in either HS578T or MDA-MB-231 cells. On the contrary, SU11274, as a single agent, significantly reduced the number of surviving colonies. Consistent with MTT assay, gefitinib/SU11274 combination reduced the colony formation in both cell lines.

The effect of gefitinib/SU11274 combination on the cell cycle distribution was further analyzed. Cells were treated with drugs for 24 h and the cells, both attached and floating, were collected to determine the cell cycle. As shown in Fig. 4B, gefitinib alone could not significantly affect the cell cycle distribution of HS578T and MDA-MB-231 cells. However, treatment of SU11274 markedly induced the accumulation of G2 accompanying by reduction of both G1 and S phase in both cell lines. The cell cycle distribution induced by SU11274 was sustained in the cells which were treated with gefitinib/ SU11274 combination.

To detect apoptotic cell death, we further analyzed the Poly (ADP-ribose) polymerase (PARP) cleavage and the level of X-linked inhibitor of apoptosis protein (XIAP) by western blot analysis. HS578T and MDA-MB-231 cells were treated with compounds for 24 h and the lysates were subjected to western blot analysis. As shown in Fig. 4C, no apparent induction of PARP cleavage was observed. The level of XIAP protein was also marginally reduced by gefitinib/SU11274 combination.

### Combination of gefitinib/SU11274 synergistically reduces the level of RPS6 in MSL subtype TNBC cells

**Figure 5:**
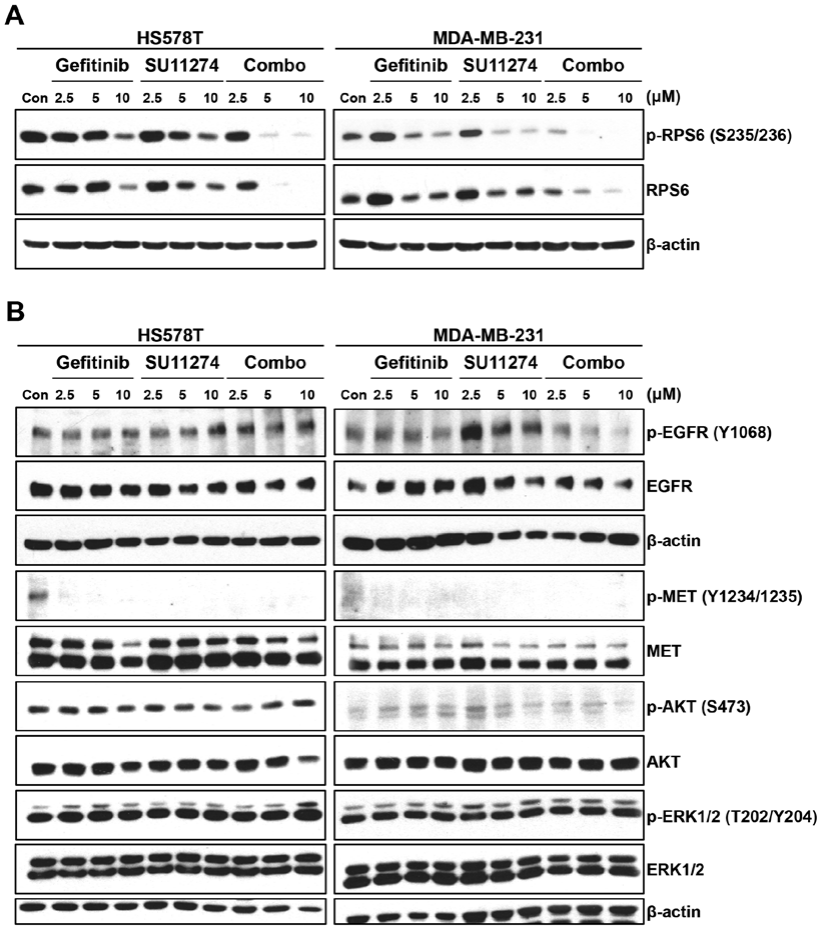
Gefitinib/SU11274 combination reduces the level of phospho-RPS6 (S235/236) and RPS6 in TNBC cells. (A and B) Cells were treated with increasing amounts of compounds as indicated for 24 h and western blot analysis was performed with indicated antibodies. β-actin was used as a loading control.

To determine signaling pathways mediating the gefitinib/SU11274 effect, we performed a series of western blot analyses. HS578T and MDA-MB-231 cells were treated with increasing concentrations of drugs for 24 h, either single agents or combination, then the lysates from these cells were subjected to western blot analysis. Interestingly, single agent treatment, either gefitinib or SU11274 for 24 h, reduced the level of phospho-ribosomal protein S6 (RPS6) (S235/S236) in HS578T and MDA-MB-231 cells in a dose-dependent manner (Fig. 5A). In addition, gefitinib/SU11274 combination synergistically reduced the level of phospho-RPS6 in these cells. Surprisingly, the level of RPS6 protein itself was reduced by these drugs as single agents and further reduced by combination treatment.

Unexpectedly, 24-h treatment of gefitinib did not reduce the level of phospho-EGFR (Y1068) in these cells, while gefitinib/ SU11274 combination reduced the level of phospho-EGFR (Y1068) only in MDA-MB-231 cells (Fig. 5B). As expected, SU11274 reduced the level of phospho-MET (Y1234/Y1235) in these cells (Fig. 5B). In addition, the level of phospho-MET was also reduced by gefitinib in both cell types. However, neither gefitinib nor SU11274 could reduce the levels of phospho-AKT (S473) and phospho-ERK1/2 (T202/Y204). The gefitinib/SU11274 combination could not reduce either phospho-AKT or phospho-ERK1/2 (Fig. 5B). These results suggest that 24-h treatment of gefitinib or SU11274 could not inhibit the AKT and ERK pathways in these cell lines.

### Gefitinib/SU11274 combination reduces the level of RPS6 in a proteasome-independent manner

**Figure 6:**
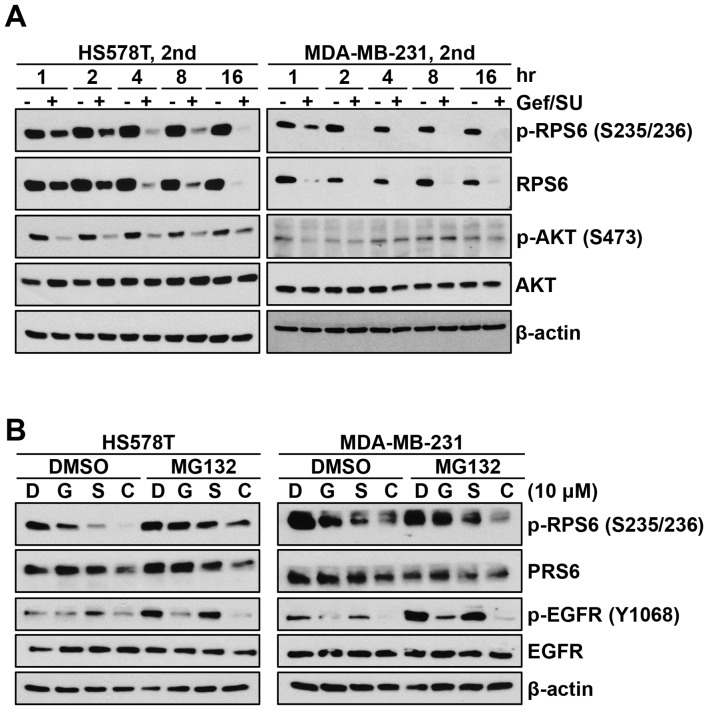
The level of RPS6 is reduced by gefitinib/SU11274 combination in a proteasome-independent manner. (A) HS578T and MDA-MB-231 cells were treated with either DMSO or gefitinib/SU11274 combination (10 μM, respectively) for indicated time and western blot analysis was performed with indicated antibodies. β-actin was used as a loading control. (B) HS578T and MDA-MB-231 cells were treated with compounds as indicated in the absence or presence of 10 μM of MG132 for 4 h and western blot analysis was performed with indicated antibodies. β-actin was used as a loading control.

To determine the level of RPS6 over time, cells were treated with gefitinib/SU11274 combination for several time intervals and the level of RPS6 proteins was detected by western blot analysis (Fig. 6A). Interestingly, the decrease of both phospho-RPS6 (S235/236) and RPS6 itself was evident as early as 1 h after treatment (Fig. 6A). In addtion, the decrease of RPS6 protein level was sustained for up to 16 h. The level of phospho-AKT (S473) was decreased at 1 h after combination treatment. However, the decrease of phospho-AKT (S473) was reversed over time in both cell lines (Fig. 6A).

The effect of proteasome inhibition on the level of RPS6 was also determined by western blot analysis (Fig. 6B). Cells were treated with 10 μM of either gefitinib or SU11274 and combination of both drugs for 4 h in the presence of the proteasome inhibitor MG132. Consistently, gefitinib/SU11274 combination markedly reduced the level of RPS6 in both cell lines. However, the treatment of MG132 did not affect gefitinib/SU11274-mediated reduction of RPS6 (Fig. 6B). Contrary to 24-h treatment, 4-h treatment of 10 μM gefitinib reduced the level of phospho-EGFR (Y1068) in these cells. In addition, gefitinib/SU11274 combination further reduced the level of phospho-EGFR. These results suggest that gefitinib/ SU11274 combination induces irreversible reduction of RPS6 in a proteasome-independent manner.

### Knockdown of RPS6 reduces the proliferation of TNBC cells

**Figure 7:**
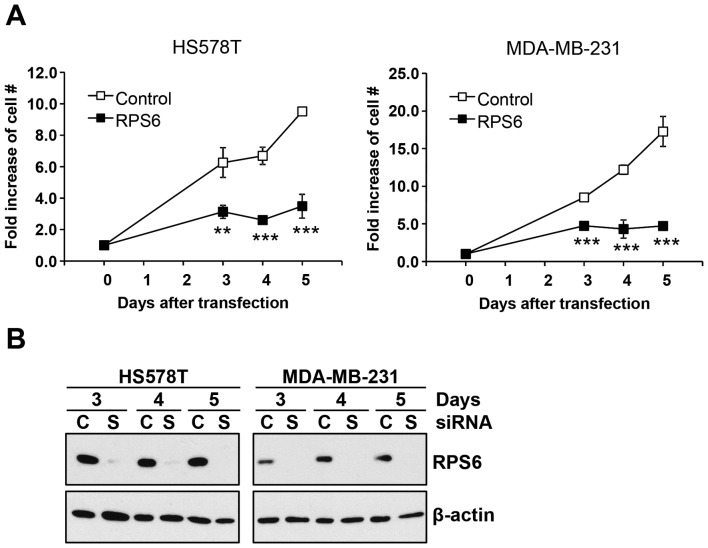
Knockdown of RPS6 reduces the proliferation of TNBC cells. (A) HS578T and MDA-MB-231 cells were transfected with either control- and RPS6-siRNA as described in Materials and methods and the number of viable cells was determined by counting viable cells with AO/PI staining. Representative data are presented as mean ± SEM from two independent experiments performed in triplicate. ^**^P<0.01; ^***^P<0.001. (B) Cells were transfected with siRNAs as described (A) and western blot analysis was performed with indicated antibodies. β-actin was used as a loading control.

Since gefitinib/SU11274 combination synergistically reduced the level of RPS6 in MSL subtype TNBC cells, we questioned whether RPS6 is important to the proliferation of these cells. To address this, we knocked down the RPS6 protein by specific siRNA. HS578T cells and MDA-MB-231 cells were transfected with either control- or RPS6-siRNA and cultivated for up to 5 days. The difference of cell proliferation was traced by viable cell counting at indicated days. As shown in Fig. 7A, knockdown of RPS6 profoundly reduced the proliferation of both HS578T and MDA-MB-231 cells as early as 3 days after siRNA transfection. Western blot analysis confirmed the knockdown of RPS6 under these conditions (Fig. 7B). Taken together, our data suggest that gefitinib/SU11274 combination reduced the proliferation of a subset of TNBC cells through downregulation of RPS6 proteins.

## Discussion

In the present study, we demonstrated that the MET inhibitor SU11274 is a synthetic lethal agent in the combination with EGFR inhibitors for the MSL subtype of TNBC cells. The levels of EGFR and MET are highly elevated in TNBC cells tested. Nevertheless, EGFR inhibitors (gefitinib, lapatinib, and BMS-599626) and SU11274 has limited potency in TNBC cells of MSL subtype such as HS578T, MDA-MB-231, and MDA-MB-436 in MTT assay. However, the combination of these drugs markedly reduced the viable cells in MTT assays and survival of these cells in clonogenic assays. One notable feature of these combinations is the reduction of RPS6 protein levels. Treatment of gefitinib/SU11274 combination for 24 h did not affect various signaling pathways including AKT and ERK. However, the level of phospho-RPS6 (S235/236) was synergistically reduced by this combination. The reduction of phospho-RPS6 (S235/236) was due to the reduction of RPS6 protein itself as early as 1 h after combination treatment. Although the level of phospho-AKT (S473) was reduced by this combination in early time points, it was reversed over time and near completely recovered at 16 h after treatment. On the contrary, the initial reduction of RPS6 protein level was maintained over time. Proteasome inhibition did not reverse the reduction of RPS6 by gefitinib/ SU11274 combination. Interestingly, siRNA-based knockdown of RPS6 itself was enough to reduce the proliferation of HS578T and MDA-MB-231 cells. Taken together our data suggest that dual inhibition of EGFR and MET induces synthetic lethality in a subtype of TNBC cells through downregulation of RPS6 protein.

MET, a member of receptor tyrosine kinase, is activated by hepatocyte growth factor/scatter factor (HGF/SF) (40). The binding of HGF to MET activates various signal pathways including RAS/MAPK, PI3K/AKT, SRC and STAT3/5 and these signal pathways mediate normal cell proliferation, cell scattering, invasion, migration, embryogenesis, evading apoptosis, angiogenesis, and tissue regeneration (41). MET and HGF are highly expressed in a wide variety of cancers including lung, ovary, renal, gastric, pancreas, head and neck and colon cancers and are also considered to contribute to unregulated cell proliferation, reduced apoptosis, altered cytoskeletal function, tumor cell scattering, migration, dissemination, and invasion during cancer cell metastasis (41–43). However, the roles of MET in the proliferation and/or survival of TNBC cells is largely unappreciated. A recent study demonstrated that paracrine activation of MET by fibroblast-secreted HGF induces gefitinib resistance in two TNBC cell lines, SUM102 and SUM149PT (44). It has also been reported that MET is colocalized with AXL receptor kinase complex which includes EGFR, HER2/3, MET and platelet-derived growth factor receptor β (PDGFRβ) in TNBC cells (45). Inhibition of MET was also reported as a potential opportunity of Notch targeting for TNBC patients with MET overexpression and Notch hyper-activation (46). More recently, the MET inhibitor PHA-665752 with the EGFR inhibitor erlotinib was demonstrated to reduce the viability of the BL1 subtype TNBC MDA-MB-468 cells (47). In the present study, we found that 10 μM treatment of the MET inhibitor SU11274 exhibited a limited potency toward MSL subtype TNBC cells in MTT assay, while 24-h treatment of 10 μM SU11274 showed significant reduction of TNBC cell survival in clonogenic assay. Interestingly, SU11274 alone induced significant increase of cells in G2 phase of cell cycle. These results suggest that MET itself might have potential role in the regulation of the cell cycle and/or long-term survival of MSL subtype TNBC cells. Further study will be needed to decipher the role of MET in the proliferation and/or survival of TNBC cells.

Our present data suggest that co-targeting EGFR and MET trigger an irreversible reduction of RPS6 protein: while the inhibition of upstream signaling pathway such as EGFR and AKT was reversed with time, the initial reduction of RPS6 protein level was sustained. In addition, knockdown of RPS6 itself significantly reduced the proliferation of MSL subtype TNBC cells in the present study. These results suggest that reduction of RPS6 by gefitinib/SU11274 combination is sufficient to inhibit the proliferation of MSL subtype TNBC cells. RPS6 protein is evolutionarily conserved from yeast to vertebrate and indispensable for protein synthesis (48). Despite the fact that increased phosphorylation and mRNA upregulation of RPS6 has been reported in several human cancers (49–61), the role of RPS6 in cancer initiation and/or progression has not been well appreciated. Recently, the potential implication of RPS6 in human cancer was revealed by knockdown experiments. Knockdown of RPS6 by siRNA reduced the survival of Ewing family tumor cell lines with near complete cell death in a siRNA library screening (62). Knockdown of RPS6 by shRNA was also reported to reduce the proliferation of diffuse large B-cell lymphoma (DLBCL) cell lines (57). Phospho-RPS6 has been reported to attenuate KRAS-induced DNA damage in acinar cells and in acinar-to-ductal metaplasia (ADM) and p53-mediated tumor suppression during initiation of pancreatic cancer (63). Collectively, targeting RPS6 may provide alternative therapeutic regimen to treat human cancers with high level of RPS6.

RPS6 protein is phosphorylated at multiple sites by various upstream kinases such as RPS6 kinase α1 (RPS6KA1), RPS6KA3, death-associated protein kinase 1 (DAPK1), and PAS domain containing serine/threonine kinase (PASK) (64–69). The phosphorylation of RPS6 is involved in the regulation of global protein synthesis that determines the size of cells, cell proliferation, and glucose homeostasis (48). RPS6 is also known to be important to cap-dependent protein translation (67). Unfortunately, the regulation of RPS6 protein stability has not yet been explored. Heat shock protein 90 (HSP90), a molecular chaperone, binds to RPS6 (70) and regulates its degradation through ubiquitin-dependent proteolysis (71). In addition, the HSP90 inhibitor geldanamycin reduces the level of RPS6 (71).

In the present study, the reduction of RPS6 by gefitinib/ SU11274 combination was not reversed by the proteasome inhibitor MG132. Since the reduction of RPS6 itself was as rapid as 1 h after treatment, it is plausible that active proteolysis regulates the gefitinib/SU11274-mediated reduction of RPS6 level. Further study is needed to address how RPS6 stability is regulated by this combination in TNBC cells.

## Figures and Tables

**Table I:** MET inhibitors identified in this study.

Inhibitor	Other name	Known targets (IC_50_ in nM)	(Refs.)
PF-2341066	Crizotinib	MET (4), ALK (24)	(72)
MGCD265		MET (1), VEGFR1/2 (3) VEGFR3 (4), RON (2), TIE2 (7)	(73)
PHA-665752		MET (9)	(41)
SU11274		MET (10), RON (4000)	(39)
XL880	Foretinib EXEL-2880 GSK1369089	MET (0.4), RON (3), VEGFR2 (0.86), FLT1 (6.8), FLT4 (2.8), KIT (6.7), FLT3 (3.6), PDGFRα (3.6), PDGFRβ (9.6), TIE2 (1.1), FGFRI1 (660)	(74)
XL184	Cabozantinib	Met (1.3), VEGFR2 (0.035), KIT (4.6), FLT1 (12), FLT3 (11.3), FLT4 (6), Tie2 (14.3), Ret (4)	(75)
